# Physiotherapy for injured workers in Canada: are insurers’ and clinics’ policies threatening good quality and equity of care? Results of a qualitative study

**DOI:** 10.1186/s12913-018-3491-1

**Published:** 2018-09-03

**Authors:** Anne Hudon, Matthew Hunt, Debbie Ehrmann Feldman

**Affiliations:** 1 Faculty of Medicine, School of Rehabilitation, University of Montreal, Pavillon du Parc, office 402-27, C.P. 6128, Succ. Centre-ville, Montréal, Québec H3C 3J7 Canada; 2 Centre for Interdisciplinary Research in Rehabilitation of Greater Montreal (CRIR), Montréal, Canada; 3 Institut de Recherche en Santé Publique de l’Université de Montréal (IRSPUM), Montréal, Canada; 4 School of Physical and Occupational Therapy, McGill University, Hosmer House, Room 205, 3630 Promenade Sir William Osler, Montréal, Québec H3G 1Y5 Canada; 5 Department of Physical Therapy, School of Rehabilitation, Faculty of Medicine, University of Montreal, Pavillon du Parc, C.P. 6128, Succ. Centre-ville, Montréal, Québec H3C 3J7 Canada

**Keywords:** Physical therapy, Musculoskeletal injury, Workers’ compensation, Qualitative research, Policy, Interpretive description, Canada, Equity

## Abstract

**Background:**

In recent years, significant efforts have been made to improve the provision of care for compensated injured workers internationally. However, despite increasing efforts at implementing best practices in this field, some studies show that policies overseeing the organisation of care for injured workers can have perverse influences on healthcare providers’ practices and can prevent workers from receiving the best care possible. The influence of these policies on physiotherapists’ practices has yet to be investigated. Our objectives were thus to explore the influence of 1) workers’ compensation boards’ and 2) physiotherapy clinics’ policies on the care physiotherapists provide to workers with musculoskeletal injuries in three large Canadian provinces.

**Methods:**

The Interpretive Description framework, a qualitative methodological approach, guided this inquiry. Forty participants (30 physiotherapists and 10 leaders and administrators from physiotherapy professional groups and workers’ compensation boards) were recruited in British Columbia, Ontario and Quebec to participate in an in-depth interview. Inductive analysis was conducted using constant comparative techniques.

**Results:**

Narratives from participants show that policies of workers’ compensation boards and individual physiotherapy clinics have significant impacts on physiotherapists’ clinical practices. Policies found at both levels often place physiotherapists in uncomfortable positions where they cannot always do what they believe to be best for their patients. Because of these policies, treatments provided to compensated injured workers markedly differ from those provided to other patients receiving physiotherapy care at the same clinic. Workers’ compensation board policies such as reimbursement rates, end points for treatment and communication mechanisms, and clinic policies such as physiotherapists’ remuneration schemes and restrictions on the choice of professionals had negative influences on care. Policies that were viewed as positive were board policies that recognize, promote and support physiotherapists’ duties and clinics that provide organisational support for administrative tasks.

**Conclusion:**

In Canada, workers’ compensation play a significant role in financing physiotherapy care for people injured at work. Despite the best intentions in promoting evidence-based guidelines and procedures regarding rehabilitation care for injured workers, complex policy factors currently limit the application of these recommendations in practice. Research that targets these policies could contribute to significant changes in clinical settings.

**Electronic supplementary material:**

The online version of this article (10.1186/s12913-018-3491-1) contains supplementary material, which is available to authorized users.

## Background

In recent years, significant efforts have been made to improve the provision of care for compensated injured workers internationally. Medical evidence-based guidelines have been developed to guide and support healthcare professionals in delivering high standards of care for these patients [1–3]. Some of these guidelines have received strong support from medical associations and insurers and have been widely discussed in order to promote their use by medical professionals [4, 5]. Recent publications have also identified and clarified key elements that could facilitate the implementation and the uptake of best practices in clinical settings in order to return injured workers to their jobs safely and in a timely manner [6, 7].

Medical doctors are not the only ones targeted by these efforts. Allied health professionals responsible for injured workers’ care such as physiotherapists and chiropractors have also developed and published position papers and best practices articles to help improve care for injured workers [8–12]. Increased attention towards factors that could facilitate or hinder the care of injured workers has also been noted [13–15].

However, despite increasing efforts at implementing best practices in this field, policies overseeing the organisation of care for injured workers can have perverse influences on healthcare providers’ practices and can prevent workers from receiving the best care possible. For example, a recent Australian study showed that general practitioners are reluctant to treat injured workers because of the current organisation of care and workers’ compensation policies [16]. In Canada, Lippel and collaborators [17] and MacEachen and collaborators [18] have also demonstrated that systemic problems can alter professionals’ work and the care they provide to injured workers. Such studies are crucial since they help clarify the various ways in which policies can modulate clinical practices and healthcare professionals’ behaviours. They can further help target the most effective strategies to improve care.

Most studies published in this area are concerned with policy influences on primary care physicians’ practices. Despite the important place occupied by allied health professionals (e.g. physiotherapists, chiropractors) in the care of injured workers, policy influences on allied health professionals’ work and practices have still been overlooked.

After physicians, physiotherapists are the second most frequently consulted professionals by injured workers in Canada. Yet, the influence of state level (workers’ compensation boards) and organisational-level (clinics or departments) policies on physiotherapists’ work and practices have not been specifically investigated. Thus, the objectives of this study were to explore the influence of 1) workers’ compensation boards’ policies and 2) physiotherapy clinics’ policies on the care physiotherapists provide to workers with musculoskeletal injuries in three Canadian provinces: British Columbia, Ontario and Quebec.

### The concept of “policy”

In our project, we investigated policies from two categories of governing bodies: workers’ compensation boards (WCBs) and physiotherapy (PT) clinics. For WCBs, policies have been conceptualised as the rules derived from provincial laws that are developed and enforced by administrators responsible for PT care at the WCB. These rules regulate the fees paid to physiotherapists and what they can or cannot do when providing care to injured workers. Policies are often described at length in official procedural documents produced for healthcare providers by the WCB, and are usually available on their website. Policies at the clinic level are more diverse and may be explicit and formalized, or tacit and informal [19]. They can be described in a clinic’s work agreement contract for professionals or in its policies and procedures manuals. Clinic policies can serve as guidelines to set expectations for employee behaviour, creating clear links with the values, mission and goals of the organization and to guide decision-making and day-to-day procedures [20].

### Choice of jurisdiction and policy overview of physiotherapy care

This study was carried out in the three most populous Canadian provinces: British Columbia (BC), Ontario and Quebec. These provinces have similarly designed compensation systems [17, 21]. However, they have implemented different models of care and WCB policies, with implications for professional practice and patient care. The three WC systems are “no- fault” systems: access to compensation is available regardless of proof of fault and regardless of legal liability of the employer [22, 23]. In each province, injured workers’ claims are administered by a para public administrator financed by employer premiums. In all three, the acceptance of an injured worker’s initial claim is determined by the WCB. However, once the claim is accepted, in both BC and Ontario, case managers are responsible for making final decisions for each patient (e.g., granting PT extensions, or for starting or modifying the return-to-work process) and health professionals’ recommendations are not binding. In Quebec, treating physicians are the primary decision makers. Their treatment recommendations are binding for other health professionals (including physiotherapists, even if these professionals remain in charge of their day-to-day treatment plan) and for the WCB, although the recommendations may be disputed through a formal procedure (8). In Quebec, doctors determine the patient’s diagnosis and prescribe the type and duration of treatment, the date of maximum medical recovery, as well as establishing functional limitations and degree of permanent impairment.^1^ Other differences related to remuneration, treatment parameters and administrative requirements that are more specific to PT care are described in Table 1 (BC [24], Ontario [25] and Quebec [26]).

**Table 1:** Key features of WCB policies for PT care

	British Columbia	Ontario	Quebec
Payment model	Block care model^1^ [Care is provided as a predetermined block of services in weeks and payment is provided for the whole block]	Program of care (POC) model^2^ • Low back • Shoulder • Musculoskeletal [Care is provided as a predetermined block of services in weeks] OR Fee-for-service (if patient is not eligible for a POC)	Fee-for-service model [Physiotherapists are reimbursed separately for each treatment session provided to the injured worker]
Length of treatment	Standard block: • 7 days for evaluation • 6 weeks of treatment Post-surgical block: • 7 days for evaluation • 8 weeks of treatment For both blocks, there is a possibility for a four-week extension if requested at least seven days before the end of the standard or post-surgical treatment block	Programs of care: • Low back POC: 8 weeks • Shoulder POC: 8 weeks • Musculoskeletal POC: 8 weeks For the fee-for-service model, the length of treatment is not specified	No pre-defined or maximum length of treatment but patient needs to see his or her treating physician after either: • 8 weeks of PT • 30 PT visits
Minimum treatment sessions per week	2 sessions per week [Fewer than two visits per week may be appropriate if the injured worker has either returned to work or is actively participating in a return to work plan, but this must be approved by the WCB]	Depending on the POC: • Low back: A minimum of 3 visits must be provided within the first 4 weeks of the program • Shoulder: A minimum of 7 visits must be provided during the 8 week program • Musculoskeletal: A minimum of 6 visits must be provided during the 8 week program	No requirements
Contact with the employer	Required by the WCB (by phone) during the evaluation period	Strongly recommended by the WCB (by phone or by a written form)	Physiotherapists usually do not communicate with patient’s employer
Clinical evaluation requirements	Physiotherapists must complete a functional evaluation that aligns with their patients’ work tasks and critical job demands	Physiotherapists are required to use a functional outcome measure to track the functional improvements of their patients, which is set by the WCB and differs for each POC	No requirements
Guidelines with regards to clinical modalities to use	None provided, treatment is left to the physiotherapist’s judgment	Yes, described in each POC manual and based on evidence	None provided, treatment is left to the physiotherapist’s judgment

^1^New model of care as of May 2014

^2^Eligibility to a POC is assessed by the injured worker’s primary healthcare provider (who may be a physiotherapist) following the criteria set by the WCB. It must also be approved by the WCB

## Methods

*Interpretive Description* guided this inquiry [27]. This methodological framework is grounded in constructivist and naturalistic approaches to qualitative research [28]. For our study, this approach was useful in developing knowledge about a domain of human experience related to health with the goal of informing professional practice [29]. *Interpretive Description* illuminated the provision of PT care for injured workers and supported the development of concrete policy recommendations applicable to current practices. More precisely, this framework supported the gathering of rich contextual knowledge about the studied phenomenon by exploring its tacit, subjective and experiential aspects from the perspective of the people involved in it, in order to better grasp its complexity and illuminate new ways of understanding it [27]. Interpretive Description was also useful for creating robust and meaningful research findings by aligning qualitative inquiry with “the epistemological underpinnings of the applied disciplines for which it is being used” [27]. In this particular research, our objective was to better understand policy factors that influence the provision of PT care for injured workers.

### Recruitment process

Two groups of participants were recruited using a purposive sampling strategy: 1) Licensed physiotherapists working with injured workers (in Quebec this included physiotherapists and PT technicians^2^) who provided information on the clinical aspects of PT care for injured workers; and 2) leaders and administrators from PT associations, professional colleges or WCBs who are not necessarily physiotherapists, but who are knowledgeable about PT policies for injured workers and who provided insight into the current macroscopic context of PT provision of care for this clientele. We used four strategies for recruitment between December 2013 and March 2015. Information about the research project was first distributed using the listservs and online bulletins of the three provinces’ PT associations and/or professional colleges. Additional potential participants were identified through the professional networks of the research team. After each interview, participants were invited to suggest others who might be interested in participating. Finally, as data collection progressed theoretical sampling was used to seek out additional participants who could speak to two areas of the analysis that were initially underdeveloped relating to the influence of the clinical setting on physiotherapists’ experiences of providing care: large private clinics that were more corporate in their orientation, as well as the perspectives of physiotherapists working in interdisciplinary teams.

A diverse set of participants was sought based on the following characteristics: role (PT clinicians, leaders and administrators), gender, practice setting (private or public; PT association, PT college or WCBs), extent of clinical experience, extent of experience treating injured workers (at least 6 months of work with injured workers), clientele (acute vs chronic patients) and location (urban vs rural, regional distribution within province). Individuals interested in participating in the study were invited to contact the first author by email. They were then sent a short demographic questionnaire in order to assess their eligibility. The questionnaire collected information about potential participants’ gender, age, geographic location, current work, and professional experience. Selected individuals then received an email inviting them to choose a time and date for the interview.

### Sample

Interviews were conducted with a total of 30 physiotherapists and 10 leaders and administrators. Information regarding clinician participants is presented in Table 2. The proportion of injured workers in their caseloads ranged from 2% to almost 100%. Participant characteristics broadly reflect the male/female ratio of physiotherapists in Canada (75% female) [30]. However, the median years of practice experience was higher in the province of Quebec. For the second group of participants (PT leaders and administrators), 8 females and 2 males from the three provinces were recruited. Participants were employed in PT provincial associations and colleges, other PT professional groups and WCBs.

**Table 2:** Participant demographics for physiotherapists

Participants	British Columbia	Ontario	Québec
Physiotherapists	9	9	9
Physiotherapy technicians (Quebec only)	0	0	3
Total	9	9	12
Gender			
Male	3	3	3
Female	6	6	9
Age^1^			
20–30	4	4	4
31–40	3	1	3
41–50	1	3	3
> 50	1	0	2
Practice setting			
Private	8	6	10
Public	0	2	2
Both private and public	1	1	0
Participants with adjunct administrative position (e.g., clinic owner/manager)	3	3	3
Years of practice as a PT^1^			
Less than 1	1	0	0
1–10	6	6	5
11–20	0	2	2
> 20	2	0	5
Years worked with injured workers^1^			
Less than 1	1	0	0
1–10	6	5	6
11–20	1	3^2^	3
> 20	1	0	3

^1^Demographic info excludes one participant from Ontario who did not complete the pre-interview questionnaire

^2^Includes one participant who had worked as a kinesiologist with injured workers prior to becoming a physiotherapist

### Data collection

All participants took part in an in-depth, semi-structured interview that was conducted face-to-face, by Skype or by phone, at a time and location that was convenient for each participant. The clinician interview guide was developed for this study based on issues identified through two focus groups with PT professionals from Quebec [14] (see Additional file 1). After being pilot tested in December 2013 with one participant, it was subsequently revised for flow and clarity. A different guide was developed for the interviews with PT leaders and administrators (see Additional file 2). Interviews were conducted by the first author in French or English depending on the preference of the participant between January 2014 and March 2015, and lasted between one and two hours (mean: 1.5 h). Seven interviews were conducted by phone, eight in person and 25 by Skype. All interviews were digitally recorded and professionally transcribed. A synopsis was prepared for each interview. WCB policy documents relative to the provision of PT care for injured workers in each of the three provinces were also collected from the respective WCB websites. They were used as a supplementary data source.

### Data analysis

Data analysis was initiated concurrently with data collection, as soon as transcriptions of early interviews were available. This iterative approach allowed us to test insights and ideas from the analysis of earlier interviews in subsequent interviews. Constant comparative methods were used to create links and to identify patterns across the whole set of data [31]. The first author coded segments of data using labels that emerged through asking questions such as “what’s going on here?” and “what does this mean?” to the data. This process was undertaken using NVivo 10 software. Different strategies were then used to condense the empirical material into broader categories. The first author created conceptual maps, diagrams and comparative tables to identify common patterns across and within data sources [31]. In collaboration with the co-authors, higher order analytic themes that addressed key elements of WCBs’ and clinics’ policies affecting physiotherapists’ provision of care for injured workers were developed. During the analysis stage of the project, reflective memos written by the first author [32] and WCB policy documents related to PT care were used as secondary data sources to contextualise the analysis of interviews and to inform the creation of the interpretive description. Throughout the analysis process, divergent as well as shared perspectives among the participants were identified, and attention was paid to negative, contradictory or outlier perspectives [27]. Provisional study results were presented and discussed in a focus group with seven physiotherapists in March 2016. The feedback from this session was used to further refine the analysis. The data collected for this study was also analysed to investigate the ethical tensions and challenges experienced by physiotherapists as they sought to live out their professional values while caring for injured workers. These results are presented in a separate publication.

## Results

Narratives from participants show that policies of WCBs and individual PT clinics have significant impacts on physiotherapists’ clinical practices. Policies found at both levels often place physiotherapists in uncomfortable positions where they cannot always do what they believe to be best for their patients. Because of these policies, treatments provided to compensated injured workers markedly differ from those provided to other patients receiving PT care at the same clinic.

Some of these differences are intended to provide an intervention that is more focused on return to work. However, other differences appear to have a detrimental effect on care quality.

The results are divided into two sections. First, we present findings related to the influence of WCB policies on PT care for injured workers. We then discuss how policies developed in PT clinics modulate PT care provision. Selected verbatim quotations are included in the results section to illustrate aspects of our analysis.

### State level workers’ compensation policies

#### End points for treatment: struggling with rigid structures of care

Participants from BC and Ontario mainly discussed how their WCBs’ models of care were restrictive in terms of *duration of treatment.* Many participants mentioned that the prescribed duration of treatment was often unrealistic for patients with complex issues. A participant said:

*You know, there’s so much variability between different types of low back issues that I don’t think it’s reasonable to expect everyone … with an acute, low back injury to improve within the 8 weeks that the program is designed for. P6B*

Several participants felt that they were pressured to get patients back to work prematurely. A majority of participants from BC perceived the “extension block” (i.e. period of treatment prolongation of a specific length granted by the WCB) as a necessary feature of their provincial WCB policy. A participant from Ontario reported being surprised that his WCB’s policies provided physiotherapists with bonus remuneration when patients return to work before the set deadline. He believed this policy could induce professionals to pressure patients into returning to work before they are ready to do so. Participants from Quebec spoke positively about the fact that the Quebec WCB does not prescribe a set duration of treatment or a set amount of PT visits. Several participants noted, however, that this latitude could sometimes lead to physiotherapists providing more treatment sessions than necessary, potentially undermining the reputation of the profession.

Conversely, a few participants from BC and Ontario discussed the positive influence of having a set treatment duration. A participant said that this “*shift in the new model has meant that physios are thinking about treating injured workers very differently right from the first visit.” D1A* Indeed, BC and Ontario participants said that the new requirements had led them to discuss the end-point of treatment with their patients much earlier than they did previously. They also stated that working with a clear return-to-work date helped them progress their treatments toward a defined goal and fostered communication about patient expectations. A participant explained:

*So…and like we let them know right from the get-go when the end date is…of like treatment, that they have 6 weeks from this date, so it’s not…it’s not ambiguous. Like they know right from the get go that this ends at a certain point. P3A*

A participant from Ontario said that this end-point prevented physiotherapists from over treating patients:

*I think if you’re treating somebody for 5 months you are not doing your job. Or they should be in a chronic pain management program, or referred somewhere else. So I think a lot of physiotherapists do a disservice to our profession by treating somebody for 10 months and not making a change. P1B*

#### Treatment requirements: dealing with imposed clinical features

Participants also discussed some specific treatment requirements set by their WCB. Ontario participants discussed features of the three programs of care (POCs).^3^ Most of these participants viewed the clinical guidelines and evidence-based modalities included in the POCs as helpful for guiding their provision of care. However, several reported that they did not always comply with them, preferring to use their own clinical experience, knowledge and judgment to plan treatments. Regarding the prescriptive nature of these programs, one participant said:

*I think that it’s great to say this is the ideal and this is how we want to treat these problems but again, there is, there’s so many other factors that may influence when and how the person is going to be able to return to work and the accommodations that the employer can make to get that person returned to work. So I think it puts a lot of pressure on, on the healthcare team to…to…work within this guideline um, knowing that there are so many other factors that may influence the success of the program. D1B*

BC participants also discussed two new features of the block care model^4^: the requirement to speak to their patients’ employer and the requirement to complete a comprehensive functional evaluation of each patient. The first requirement was viewed differently. Some mentioned that it allowed for a better understanding of patients’ working environment and positioned them to make more pertinent recommendations to the WCB regarding return-to-work. Others were uncomfortable with this task, fearing to compromise patient confidentiality and their trust relationship with patients. Most participants viewed the functional evaluation as relevant and useful to help their patients progress towards their return to work. Again, a few participants felt they were not adequately prepared to perform such functional evaluations. In that regard, a participant said:

*I think one of the challenges with this new system is that they’re asking the physiotherapist to venture into things that not necessarily every physiotherapist has experience with and then I’m particularly referring to two things. One would be doing some specific functional and fitness testing with somebody…particularly the functional testing, and then being able to relate that and make the connection with what peoples’ job demands are. P9A*

Nevertheless, most participants saw these new clinical requirements as demonstrating the WCB’s trust in their clinical competencies.

#### PT reimbursement rates: failing to appreciate professionals and workers’ value

PT reimbursement by WCBs was a concern of almost all participants. As insurers, provincial WCBs determine the fees paid to PT professionals for treating injured workers. In all three provinces, WCBs have set remuneration fees and clinics are prohibited from charging a co-payment fee to injured workers. WCB fees mostly affected two types of participants: self-employed physiotherapists (including contractors working in PT clinics) and clinic owners/managers. Self-employed or contractual participants explained that they usually receive a percentage of the fee paid to their clinics. Since WCB fees for treating injured workers are lower than what they receive when treating other clienteles (regardless if it’s fee-for-service or a block care model), these participants receive less money when treating injured workers:

*[…] my private patients pay $70 a visit for half an hour, of which I get 60%. WCB pays, […], if you average out the visit cost it would be $60 a visit of which I get 50%. So it’s quite a fair big difference for me. P5A*

Many clinic owners and/or managers also said that the fees set by WCB for PT services negatively affected their clinic’s revenue. WCB fees were most likely to be perceived as “too low” by the Quebec participants, although participants from all provinces discussed this issue. Participants also discussed ways in which the imposition of low fees can influence the care provided to patients. Indeed, some participants said they preferred not to treat or to treat only a small number of injured workers because of the loss in salary associated with treating this clientele. The low fees paid by WCBs also seemed to contribute to the stigmatization of injured workers since some participants identified them as patients who do not pay well. The dissatisfaction regarding WCB fees by clinic owners and managers in BC, Ontario and Quebec led to enforcement of certain policies by PT clinics, which are described in greater detail later in this article.

#### Communication mechanisms: managing disconnected parties

Administrative requirements regarding communication between physiotherapists and WCBs were a major topic of discussion. In all three provinces, WCBs require physiotherapists to complete standardized administrative reports that are sent to the WCB on a predetermined basis. These reports are used to assess the patient’s clinical progression during the rehabilitation process. WCBs also require physiotherapists to communicate with them by phone to inform them of any significant change in their patient’s status. The frequency and format of these communications vary greatly between provinces, and across these differences, a majority of participants explained that current ways of communication do not facilitate the provision of care for injured workers. For example, several participants from Quebec said they believed their clinical reports (completed every 3 weeks) were not read by the WCB case managers.

*That’s why I often think than when one thinks that our work is not read or recognized or taken into account, I think this is why the WCB files or progress reports are poorly completed. OK? While probably a better communication between the case manager and the physio, in terms of progress report, would improve this link. P1C*

Some suggested that this is the result of WCB case managers being too busy to read all the reports they receive. More than half of participants also talked about the difficulty of reaching case managers (playing “telephone tag”), although prompt communication is a WCB requirement. Several participants said they wished they could communicate with the WCB in a timely fashion and in more flexible ways (e.g., emails, secure Internet portal, possibility of planned phone meeting).

The lack of clarity about what to communicate to the WCB was also linked to communication policies. Some participants from all three provinces mentioned that they were uncertain what the WCB actually needed to know to adequately manage their cases and that the current forms are not effective for sharing information.

*So do they want us to report what they are having trouble with in terms of ADLs [activities of daily living], or do they want an actual “here are limitations that we want to see imposed at work?” Like those are… I think those are both very valid interpretations of what that box is asking for, um, but we don’t know what WCB is looking for. P4B*

Hence, participants mentioned the need for greater clarity in their interactions with WCBs to better respond to communication requirements.

In summary, participants discussed at length the clinical and administrative requirements from WCBs’ policies and explained how these can affect the provision of care for injured workers.

### Organizational-level policies (PT clinics)

In almost all the interviews, participants discussed the influence of their own clinical setting’s policies on the care they provide. They highlighted policies regarding treatment parameters and choice of professional (e.g., kinesiologist versus physiotherapist), physiotherapists’ remuneration schemes and support for clerical tasks. Their influence was experienced as more constraining in private PT clinics compared to those in the public sector. A participant explained:

*I would say the employers do have a role to play because I’m in a private clinic. It’s you know, they want to make money and they’ll get a lot more money from a private patient than they will from a WCB patient. So, I can see their motivation to change the way that their um, they’re scheduled and the way that they’re um … the way they handle these sorts of patients. P9B*

#### Treatment session parameters and choice of professional: Predetermining clinical care

Some clinics’ policies were described as having a negative influence on physiotherapists’ work and patient care, including policies regarding treatment session length and frequency. In Ontario and BC, some participants reported that their employer (the clinic owner) set a maximum number of treatment sessions for the whole block-care or POC model because of financial considerations:

*So there are definitely clinics that are working to the monetary outcome, before the worker outcome. So they’re seeing the worker less often than is ideal because they don’t want to lose money or they don’t want to minimize how much they’re getting paid; so some clinics, most of the big box clinics […] are quite open with saying “no, we’re only seeing workers twice a week”. D1A*

Hence, these participants explained they had no real choice about the number of sessions for these patients. A similar situation was discussed with regard to the length of each session. In order to preserve clinic profitability, participants from the three provinces said they had witnessed or worked in clinics where injured workers’ sessions were shorter (e.g., 20 min instead of 30 min) than those provided to other patients.

*I also know of some who they’ll see WCB but they will limit their treatment… uh, times… a little bit, like more than we would, so that they can, we’ll figure out, you know, if this is all WCB fee for the session, then they would only get half or a quarter of the time say that a private patient would, just because of that difference in funding. P6B*

Other participants explained that after conducting the initial evaluation of a patient, their clinic required them to transfer injured workers to a PT assistant, PT technician or to a group therapy class led by a kinesiologist so that it would be more profitable for their clinic. This was not the case for patients whose treatment was not covered by the WCB. A participant from Quebec explained:

*…it’s easier to suggest a PT technician to people who don’t have the money to pay for the service, it’s easier, it’s easily more possible to have it accepted as a modality, than a patient who comes to a private facility who says:*” *good, as for me, I pay and I want to do business with Mr or Ms X […]”. P1A*

In Ontario and BC, several participants explained that they would have preferred to carry out all aspects of their patients’ PT care without involving a kinesiologist or assistant.

*[…] if you ask me, I prefer to just… cause not that I don’t trust the assistants with exercises but I feel like I’d rather be the one who is doing it to so I know how patients respond to it and how I can progress them, basically. P3B*

In sum, although these clinic policies were sometimes described as “logical” or “fair” by participants, most said they were uncomfortable with the way their clinics systematically discriminated against injured workers: *Well ... well, it very much confronts my values that my employer sees [injured workers] as a source of money and not as people who suffer. P2C.*

#### Physiotherapists’ remuneration schemes: creating unnecessary insecurity

Clinic policies regarding physiotherapists’ remuneration also influence patient care. In BC, one participant said her clinic only pays her on receivables and not on what is actually billed by the clinic. With the new block-care model, she said she only gets paid around 6 weeks after the end of the treatment block, which creates financial insecurity. In Quebec, some participants said that paying physiotherapists “by the visit” was a clear incentive for professionals who do not have a stable caseload of patients to see injured workers more frequently, even when it is not clinically justified. This concern was often discussed in relation to younger physiotherapists without an established clientele and who are usually the ones who treat injured workers:

*I am not at the stage where I am going to keep a patient just for the extra pennies it will bring me. Because I know that when he will be discharged, there will be a new one that will come in, you know […] I don’t have this worry like the new ones who just started to work, but I understand them completely, you know, they have student debts, they have more debt to, to, to start out in life, as they say. P12C*

Participants working in public clinical settings and those who are paid an annual salary did not express these concerns.

#### Support for clerical tasks: reducing physiotherapists’ load

Some clinic policies were identified as making a positive contribution to physiotherapists’ work and facilitating the care for injured workers. Participants from all provinces discussed the positive impact of the support provided by the clinic administrative personnel when dealing with injured workers’ paperwork. Participants also expressed their satisfaction with regards to their employers when they provided paid time during the week to complete paperwork and make phone calls. Most participants who said they were not provided with time for these tasks in their schedule chose to complete them during patient treatment time:

*If my day is fully booked I have no spare time to do that but all these extra additional non-billable caseload management work has to be done before work, after work or in breaks, in my lunch. Right? So that makes a long work day so I wish my clinic paid me for that [...]. But this is my clinic’s problem, this is not WCB. P3B*

On the whole, when discussing their clinics’ policies, participants mentioned the importance of having leeway to complete their professional work with injured workers and for their employer to trust their clinical judgment. Participants who seemed dissatisfied with having to treat injured workers often worked in clinical settings that imposed more rules and tacit policies that resulted in treating injured workers differently than other patients.

## Discussion

Results from this study show that, when caring for compensated injured workers, patient-provider relationships and physiotherapist’s clinical decisions are strongly modulated by policies established at the state (WCB) and organizational levels. The physiotherapists, leaders and administrators whom we interviewed in BC, Ontario and Quebec identified many of these policies as hindering and limiting the provision of equal and quality care for injured workers. These findings are of major importance since they provide new evidence that the best clinical guidelines in the world and the best healthcare professional training won’t be sufficient to change current care problems currently witnessed by Canadian injured workers.

This notion that clinical practices are modulated on a day-to-day basis by insurers’ policies at a state level is important to acknowledge since third party payers play a significant role in financing physiotherapy (PT) care for people with musculoskeletal disorders everywhere in Canada. These payers, consisting mainly of private insurance companies, employer-based healthcare plans, car accident insurers, and workers’ compensation boards (WCB), are extensively involved in covering - in whole or in part - the costs of PT treatments not covered by the Canadian Health Act^5^ [33].

Our study was the first to explore workers’ compensation and clinics’ policies influence on injured workers’ PT care in Canada, but our results corroborate worrying findings from the United States (US) regarding PT and medical care. For example, an American study showed that policies set by Medicare aiming to rationalize and downsize rehabilitation costs influenced physiotherapists’ clinical decisions [34]. In the same vein, the establishment of a therapy threshold for PT care within the US Medicare prospective payment system (i.e., imposition of a maximum reimbursement cap of 10 sessions) led to ethical struggles and affected therapy practice patterns [35]. Cost containment approaches to care in the US by WCBs (e.g., fee schedule, provider choice limitation, managed care) have led to inferior treatment outcomes, poor quality of care and high costs [36]. A large study on workers’ compensation policies in seven American jurisdictions revealed that as insurance price control for chiropractic services increased, chiropractors tended to increase the number of services billed per visit; a strategy attributed to maintaining their income [37]. In Canada, physicians also reported that their ability to provide treatment they felt their patients required was impaired by the rigidity of WCB policies [17]. Thus, as shown by these articles and the results of our own study, the impact of third party payers’ policy structures on healthcare professional should be part of discussions about improvement of quality care for patients and be put at the forefront of deliberations.

Discussions about ethical standards of practice and their importance in providing care to injured workers have also been recently discussed in the scientific literature [38, 39] in an attempt to resolve certain problematic aspects of care. Ethically, WCBs and PT clinics have fiduciary obligations to implement policies that support fairness and claimant dignity, and that avoid stigmatization and prevent discrimination toward injured workers [21]. Even though Workers’ Compensation Acts do not explicitly articulate these requirements, they do confirm that Canadian WCBs were created following a historic compromise where injured workers surrender their right to sue their employer for their injury in exchange for a compensation system [23]. As such, and in line with the commitments WCBs publicly make to provide high quality services [40] and fair benefits [41, 42] to injured workers, they should fulfill these obligations. Results from this study show that these obligations are far from being completely fulfilled at the moment. Low reimbursement rates for physiotherapists, burdensome treatment requirements and difficulty in communication can contribute to further stigmatization of a clientele that is already dealing with important difficulties: being sick, often not working and supported by a third party payer. PT clinics’ reactions to WCB policies further amplify the problem and many clinics’ owners described by our participants seemed to have forgotten that they also have obligations to make sure that the values of quality of care and equity that lie at the core of PT profession’s identity should not be outweighed by financial considerations such as optimizing profits [43, 44]. In that regard, several participants expressed being conflicted between what they are told to do and what they believe they should do, and most of them were uncomfortable when they witnessed practices which they viewed as discriminatory or inequitable. However, few participants seemed to be aware that “*the rules of the system drive the behaviour*” [17] and did not explicitly link the practices they observed with underlying policies. Physiotherapists working with injured workers should have the opportunity to learn about the influence that formal or informal policies can have on their practice, including harmful effects. Fostering the adoption of a critical stance toward current ways of practising seems of utmost important for physiotherapists working with injured workers, but also for all professionals caring for this clientele [45–47]. Healthcare professionals should thus move from a circumscribed focus on patient treatment to become more attentive to the policy processes that shape their behaviours [48].This awareness and reflexivity could help them advocate for changes to rules and policies at different levels.

Findings from our study also demonstrate that considerable work is needed to improve PT care for injured workers. Some concrete solutions can be envisioned. Following the concept of clinical governance [49], it seems important that stakeholders involved in the process of care for injured workers, including physiotherapists, administrators or managers, insurers and politicians aim toward better integrating services for this clientele. Policies found at the clinical level in this study were often established in reaction to problematic WCB state level policies. A good start in improving patients’ care would be to develop PT policies in a collaborative and more transparent way at both levels. Policies at the WCB level are currently developed in political contexts, by politicised actors. According to participants, this process does not always involve stakeholders who have good knowledge of clinical realities in physiotherapy. Policies that are detached from the clinical context have the potential to be regarded as less relevant and overly restrictive by health professionals [49]. Insuring that a variety of stakeholders with knowledge of the work disability field, including patient representatives, physiotherapists and work disability researchers, are able to inform the development or refinement of WCB policies regarding PT care might help address current challenges reported by the participants.

Employing a physiotherapist with extensive clinical knowledge in the work disability field ‘in-house’ at the WCB might also be helpful to evaluate applicability and coordinate the implementation of improved PT policies on the provision of care in clinics, as well as creating links and increasing collaboration with PT provincial associations. Long-term collaborations between PT associations and WCBs could also be initiated so that specific challenges could be better understood by both parties and concrete solutions, adapted to the policy context of each province, developed and implemented.

WCB policies from BC and Ontario also encourage physiotherapists to speak with their patients’ employers to help plan the return to work. These policies thus push physiotherapists to account for realities of the workplace, a factor that has been shown to play a determining role in the return to work process after an injury [50] but has yet to be widely implemented in PT care [8]. Programs of care put in place by the Ontario WCB propose evidence-based guidelines to direct physiotherapists’ practice for different types of injuries. Although these guidelines (as for any practice guideline) should not be used without clinical judgement and are mostly generic, they nonetheless provide up-to-date guidance on modalities that have been proven to be effective (as well as those that are ineffective) for certain types of injuries and are supported by a clear policy at the WCB level. In addition, the Ontario WCB requires physiotherapists to use functional outcome measures adapted to each POC to evaluate the improvement of injured workers. These outcome measures possess good psychometric properties and their prescribed use insures objective measurement of the patient’s progression. Indeed, it has been shown that insurance providers can promote the use of best practices [51]. These initiatives are all good starting points in order to facilitate the alignment between best clinical requirements and policy measures at the organizational and state levels. Other initiatives that aim to create bridges between physiotherapists’ practices and policy statements should be encouraged. As such, a promising area where clinical practice should meet state level mechanisms and policies relates to the need for feedback for healthcare professionals and clinics regarding the care they provide. Indeed, health professionals and health organisations need to receive feedback on their performance so they can evaluate their practices [52] and motivate them to improve. BC WCB has recently implemented a policy to provide personalized feedback to each PT service provider by means of a confidential online report card. To our knowledge, Ontario and Quebec WCBs have no specific feedback or performance evaluation systems. The BC system should be studied and, if proven effective, its uptake should be encouraged more broadly. Furthermore, this could also be applied to other healthcare provider services for injured workers.

### Study limitations and strengths

Over the course of the interviews, we observed that several participants were hesitant or refrained from discussing certain topics, despite being reassured regarding the steps that the research team would take to preserve their anonymity. Since the physiotherapy community of leaders and administrators with regards to injured workers in Canada is small, and because some potential participants expressed concern that they could be identified based on their demographic information, we did not collect these details. Therefore, we are unable to present this information in the paper. PT care for compensated workers appears to be a sensitive and even politicised topic, especially for participants who hold management or leadership positions. Consequently, it is possible that some important information may not have been shared during the collection of data, which might limit the reach of our investigation of this phenomenon. Further, the web of systemic features involved in the provision of care for injured workers is complex and our study may have only revealed certain aspects that affected the participants. Nevertheless, our results provide interesting insights on how current policies affect injured workers’ PT treatments - an issue that has not been previously investigated. Finally, although we provided contextual details about the larger socio-political context surrounding the provision of PT care in the three provinces, it was impossible to fully address the specificities of each of the province’s workers’ compensation regulatory regimes in this article since they differ considerably.

### Future research

This study corroborates the importance of recognizing that patient care is largely influenced by the organizational dynamics of healthcare institutions and compensation structures rather than upon individual professionalism [53]. Even though this study sheds light on important concerns regarding PT care for injured workers, the influence of WCBs’ policies on PT care has yet to be comprehensively studied [54]. New studies could thus aim to further explore and understand the influence of inter-organisational interactions on the provision of PT care for injured workers, using the concept of clinical governance, for example [49]. Future studies could also use the frameworks of business and organizational ethics to investigate how administrators from WCBs and PT clinics could foster a strong ethics cultures in their organizations and insure that specific policies and procedures allow espoused values to be proactively incorporated on a day-to-day basis [53]. Other studies could also aim to quantify the impact of certain macro and meso-level policies on outcomes of care for patients and professional satisfaction. Follow-up projects could then target and modify problematic policies in order to aim for greater equity and improved care for injured workers.

## Conclusion

This study demonstrates that clinical practices and physiotherapists’ behaviours in the provision of care for injured workers in Canada are strongly influenced by policies of WCBs and PT clinics. Our results show these policies can alter the provision of equitable and quality care. At the WCB level, clinical and administrative requirements regarding treatments interventions, end points for treatment, reimbursement rates and ways of communicating can create challenges for physiotherapists and affect patient care. Provincial WCBs should acknowledge the influence their policies can have on the provision of PT care. PT clinics are for-profit entities. Nonetheless, they provide important health services and need to ensure that equitable and quality care is provided to all their clients. Clinic owners and managers should implement policies that promote equity, and critically appraise whether their policies could lead to a lower standard of care for injured workers. Physiotherapists working in the occupational health field in each province could advocate for policies that could reduce challenges encountered while treating injured workers. Findings from this study could also serve as a catalyst to further explore and understand the way state level and organizational policies could be developed to be better aligned so they could in turn facilitate the use of best practices and promote ethical and quality care for injured workers across the country.

## Additional files

Additional file 1: Summary of key questions asked to clinicians participating in the study. (DOCX 129 kb)

Additional file 2: Summary of key questions asked to leaders and administrators participating in the study. (DOCX 147 kb)

## Abbreviations

BC: British Columbia
POCs: Programs of care
PT: Physiotherapy
WCBs: Workers’ compensation boards

## Footnotes

1 An Act Respecting Industrial Accidents and Occupational Diseases, R.S.Q., c. A-3.001, (ARIAOD) s. 212.

2 PT technicians are healthcare professionals who are included under the category of PT professionals. They have a technical college diploma, in contrast to physiotherapists who have a university master’s-level training. The term “physiotherapist” is used throughout the text, but it also includes PT technicians when discussing Quebec PT professionals.

3 In POCs, PT care is provided as a predetermined block of services that last 8 weeks. These programs separate patients in three groups: low back POC, shoulder POC and musculoskeletal POC. See Table 1 for more information.

4 See Table 1 for more information about the block of care model in British Columbia.

5 PT services covered in the Canadian Health Act mainly refer to hospital-associated care such as inpatient hospital PT, PT services provided by community service centers (often observing strict criteria and usually accessible following an inpatient hospital stay) and PT provided in outpatient hospital departments (often accessible to patients who underwent a surgery or who have been referred through a special hospital program)
