# Supplementary material for: Physiotherapy for injured workers in Canada: are insurers’ and clinics’ policies threatening good quality and equity of care? Results of a qualitative study
Source: BMC Health Serv Res. 2018 Sep 3;18:682. doi: 10.1186/s12913-018-3491-1 (PMC6122715; doi:10.1186/s12913-018-3491-1)
Supplement: Supplementary file 1 — Summary of key questions asked to clinicians participating in the study. (DOCX 129 kb) [file 12913_2018_3491_MOESM1_ESM.docx]

**Interview Guide for clinicians - Main questions**

**a) Daily Practice**

- What is your role in your clinic/hospital/work setting?
- Could you tell me how the rehabilitation of patients compensated by WCB works in your clinic? What is your specific role with WCB patients?

**b) General problems encountered during treatment of WCB patients?**

- What recurrent (administrative?) problems do you encounter when treating WCB patients?
- What are some constraints you face when dealing with patients WCB?

**c) Administrative or organizational issues**

- How do you manage your time when you treat patients compensated by the WCB? (hands-on treatment with the patient, forms writing, calls, communications)
- How do you experience relationships/collaborations with: the patients, the WCB case manager, the physician, the employer, other colleagues in physiotherapy or other disciplines (massage therapy, occupational therapy, osteopathy)?
- How would you describe your knowledge of the operating system of the WCB? Is it good? Average? Why?

**d) Question of ethical issues and professional values**

- Have there been times where you witnessed unfair practices or inequalities in the course of your treatments with WCB patients? Can you describe these moments?
- Have certain problems you encountered while treating WCB patients countered your professional or personal values ​​as a physiotherapist?

**e) Financial and legal issues**

- Are there financial issues associated with treating WCB patients in physiotherapy? Do you have examples?
- Have you witnessed any conflicts of interests in your practice with patients compensated by the WCB? If so, can you give me some examples?
- Are there any regulations or requirements from the WCB that do not seem to work well in practice? What are they?

**f) Care provided to patients**

- In light of all that we just discussed, do you feel that any problems you may have identified impede the quality of treatment offered to WCB patients? If so, how?
- If you identified problems, how do you think they affect the system as a whole? Or affect the effectiveness of the system?
- Are the tools physiotherapists use to evaluate and treat the WCB patients appropriate? Why or why not?

**h) Possible solutions**

- Are there solutions that you think could improve the care offered to WCB patients in physiotherapy?
