# Supplementary material for: Physiotherapy for injured workers in Canada: are insurers’ and clinics’ policies threatening good quality and equity of care? Results of a qualitative study
Source: BMC Health Serv Res. 2018 Sep 3;18:682. doi: 10.1186/s12913-018-3491-1 (PMC6122715; doi:10.1186/s12913-018-3491-1)
Supplement: Supplementary file 2 — Summary of key questions asked to leaders and administrators participating in the study. (DOCX 147 kb) [file 12913_2018_3491_MOESM2_ESM.docx]

| **Place of physiotherapists** | - Physios’ role in the treatment of injured workers (IW) supported by workers compensation board (WCB)? - Which skills and competencies are physios providing when treating IW? Are they missing some? - What about competencies and skills aimed at facilitating return to work (RTW)? Do clinical settings have the appropriate resources? - What about the professional autonomy of physios inside the system and the WCB system? - Do physios have a good or sufficient knowledge of the WCB system and of its implications on patients’ treatment and care? |
| --- | --- |
| **Management and decisions** | - How does the rehabilitation of workers and care provided to them by physios work in your province? - How do decisions concerning provision of care are taken and by whom (consultation, collaboration with which parties)? |
| **Interprofessional collaboration** | - What about the collaboration of physios with other professionals involved in the care of IW? - What do you think of physios’ communications with these others stakeholders: MD, OT, employer and supervisor, agent or advisor of the WCB, chiropractor? |
| **Intraprofessional collaboration** | - What about the implication of PTAs in care provided to IW by physios? |
| **Constraints to care or to provision of services** | - What are the constraints to providing physio treatment to IW (compared to another clientele)? - Are there recurring or more general problems that come to your mind? |
| **Quality of care** | - What do you think of the quality of care provided to IW by physios? And of the availability of services? - What are the major obstacles to a high quality of care? |
| **Organizational issues** | - What are the institutional, organizational and professional constraints or issues concerning the treatment of IW by physios? |
| **Ethical issues** | - What are the ethical issues that physios face? - Are here any tensions between the different roles and responsibilities of physios? |
| **Stakeholder decisional role and role of his/her organization** | - What are the expectations of your organization towards physios who work with IW? - Do you play a role as ____________ (the role of the decision maker) in improving the care provided to IW by physios? - What is the role of your organization in the provision of care and in improving care for IW? |
| **Perception of the decision-maker towards his or her organization** | - What are your perceptions of your own organization and the way it deals with the treatment of IW by physios? |
| **Financial issues** | - What are the financial issues taking place in the rehabilitation of IW compensated by WCB? |
| **Creation of professional guidelines or standards** | - Are there any guidelines that can guide clinical practice with IW compensated by the WCB? - Do you think it would be useful to develop such guidelines or professional standards to help IW rehabilitation? |
| **Solutions** | - What changes should be made to improve the system? - What are the changes that should be made to improve the quality of care in physiotherapy for IW? |

**Interview Guide for leaders and administrators - Main questions**
